# Combined Effects of Ocean Acidification and Light or Nitrogen Availabilities on ^13^C Fractionation in Marine Dinoflagellates

**DOI:** 10.1371/journal.pone.0154370

**Published:** 2016-05-06

**Authors:** Mirja Hoins, Tim Eberlein, Christian H. Groβmann, Karen Brandenburg, Gert-Jan Reichart, Björn Rost, Appy Sluijs, Dedmer B. Van de Waal

**Affiliations:** 1 Department of Earth Sciences, Utrecht University, Utrecht, The Netherlands; 2 Marine Biogeosciences, Alfred Wegener Institute Helmholtz Centre for Polar and Marine Research, Bremerhaven, Germany; 3 Department of Aquatic Ecology, Netherlands Institute of Ecology (NIOO-KNAW), Wageningen, The Netherlands; 4 Geology Department, Royal Netherlands Institute for Sea Research (NIOZ), Den Hoorn (Texel), The Netherlands; GEOMAR Helmholtz Centre for Ocean Research Kiel, GERMANY

## Abstract

Along with increasing oceanic CO_2_ concentrations, enhanced stratification constrains phytoplankton to shallower upper mixed layers with altered light regimes and nutrient concentrations. Here, we investigate the effects of elevated *p*CO_2_ in combination with light or nitrogen-limitation on ^13^C fractionation (ε_p_) in four dinoflagellate species. We cultured *Gonyaulax spinifera* and *Protoceratium reticulatum* in dilute batches under low-light (‘LL’) and high-light (‘HL’) conditions, and grew *Alexandrium fundyense* and *Scrippsiella trochoidea* in nitrogen-limited continuous cultures (‘LN’) and nitrogen-replete batches (‘HN’). The observed CO_2_-dependency of ε_p_ remained unaffected by the availability of light for both *G*. *spinifera* and *P*. *reticulatum*, though at HL ε_p_ was consistently lower by about 2.7‰ over the tested CO_2_ range for *P*. *reticulatum*. This may reflect increased uptake of (^13^C-enriched) bicarbonate fueled by increased ATP production under HL conditions. The observed CO_2_-dependency of ε_p_ disappeared under LN conditions in both *A*. *fundyense* and *S*. *trochoidea*. The generally higher ε_p_ under LN may be associated with lower organic carbon production rates and/or higher ATP:NADPH ratios. CO_2_-dependent ε_p_ under non-limiting conditions has been observed in several dinoflagellate species, showing potential for a new CO_2_-proxy. Our results however demonstrate that light- and nitrogen-limitation also affect ε_p_, thereby illustrating the need to carefully consider prevailing environmental conditions.

## Introduction

Anthropogenic activities have caused the partial pressure of CO_2_ (*p*CO_2_) in the atmosphere and oceans to increase at an unprecedented rate [1]. This will shift marine carbon speciation towards increasing CO_2_ and bicarbonate (HCO_3_^-^) concentrations, and decreasing carbonate ion (CO_3_^2-^) concentration and pH [2]. Along with these changes in carbonate chemistry, global temperatures are expected to rise by 2 to 6°C within this century [1], which likely leads to enhanced (thermal) stratification for most oceanic regions [3]. Enhanced stratification can cause primary production to decrease, as observed in low-latitude oceans [4], where the mixed layer depth is already relatively shallow and upwelling of nutrient-rich deeper water masses is suppressed. Alternatively, enhanced stratification may increase primary production in regions with deep mixed layer depths, such as in high latitude oceans. At such locations, phytoplankton may be light-limited due to the deep convective turnover [5]. Irrespective of the net effect on primary production, shoaling of the thermocline causes phytoplankton to be more often restricted to the upper layers of the water column, characterized by high irradiance and low nutrient concentrations [6]. Such changes in light intensity and nutrient concentration may affect marine phytoplankton, including dinoflagellates.

Dinoflagellates are unicellular eukaryotes and can reach high densities under favorable environmental conditions, which may lead to harmful algal blooms with adverse effects not only for the aquatic food web, but also for human health (e.g. [7; 8]). Strategies that add to their success include toxin production, allelopathy, mixotrophy and cyst formation [9; 10; 11; 12]. While studies have investigated how dinoflagellates are influenced by changes in pH and/or *p*CO_2_ [13; 14; 15; 16; 17], less is known about the combined effects of CO_2_ and light availabilities (as daylength, see [18]) or CO_2_ and nitrogen-limitation [19]. Like all phytoplankton, dinoflagellates fix CO_2_ with the carboxylation enzyme Ribulose-1,5-bisphosphate Carboxylase/Oxygenase (RubisCO), which discriminates between carbon isotopes, favoring ^12^C over ^13^C (e.g. [20; 21; 22]). The inorganic carbon (C_i_) species taken up by phytoplankton differ in their isotopic composition, with CO_2_ being ^13^C-depleted compared to HCO_3_^-^. Under elevated CO_2_ concentrations, dinoflagellates may take up relatively more CO_2_, resulting in higher ^13^C fractionation (ε_p_) [23; 14]. Similarly, high CO_2_ efflux:total C_i_ uptake (i.e. leakage) prevents the accumulation of ^13^C within the intracellular carbon pool, thereby increasing ε_p_ [23; 14]. Indeed, ε_p_ values in different phytoplankton groups, including dinoflagellates, were shown to increase with elevated *p*CO_2_ [18; 24; 14; 25; 17].

Organic dinoflagellate cysts are ubiquitously preserved in marine sediments (e.g. [26]). The CO_2_ dependency of their isotopic composition may be reflected in their cysts, thus potentially providing a proxy for past CO_2_ concentrations. However, the CO_2_ dependency in ε_p_ may be affected by other environmental conditions, such as the availability of light and nutrients (e.g. [27; 28; 29; 30]). Here, we investigate the combined effects of elevated *p*CO_2_ and low-light conditions or nitrogen-limitation on particulate organic carbon (POC) production (μ_c_), Chlorophyll-a (Chl-a):POC ratios and ε_p_ in four marine dinoflagellate species. We grew *Gonyaulax spinifera* and *Protoceratium reticulatum* under low-light conditions (LL) and *Alexandrium fundyense* and *Scrippsiella trochoidea* under nitrogen-limiting conditions (LN) and compared these responses to results from an earlier study, where the same species were grown under high-light and nitrogen-replete conditions (HL and HN).

## Materials and Methods

### Experimental Set-up

For the high-light and nutrient-replete conditions, experiments were performed as dilute batches with *Gonyaulax spinifera* (strain CCMP 409), *Protoceratium reticulatum* (strain CCMP 1889), *Alexandrium fundyense* (strain Alex5, [31]; previously *A*. *tamarense* [32]), and *Scrippsiella trochoidea* (strain GeoB267; culture collection of the University of Bremen). Each strain was grown in 2.4 L air-tight borosilicate bottles at a constant temperature of 15°C and dissolved CO_2_ concentrations ranging from ~5–50 μmol L^-1^. CO_2_ levels of 180, 380, 800 and 1200 μatm were obtained by mixing CO_2_-free air (<0.1 μatm *p*CO_2_, Domnick Hunter, Willrich, Germany) with pure CO_2_ (Air Liquide Deutschland, Düsseldorf, Germany) using mass flow controllers (CGM 2000, MCZ Umwelttechnik, Bad Nauheim, Germany). Each of the *p*CO_2_ treatments was performed in biological triplicates (n = 3). Experiments were carried out at low cell densities with final concentrations <400 cells mL^-1^, ensuring negligible changes in carbonate chemistry of <3.5% with respect to dissolved inorganic carbon (DIC).

As growth medium, filtered North Sea seawater (cellulose acetate membrane, 0.2 μm pore size, Sartorius, Göttingen, Germany) with a salinity of 34 and enriched with 100 μmol L^-1^ nitrate and 6.25 μmol L^-1^ phosphate was used. FeCl_3_ (1.9 μmol L^-1^), H_2_SeO_3_ (10 nmol L^-1^) and NiCl_2_ (6.3 nmol L^-1^) were added according to K medium [33], and metals and vitamins were added according to f/2 medium [34]. Bottles were placed on a roller table in order to avoid sedimentation. Daylight tubes (Lumilux HO 54W/965, Osram, München, Germany) provided incident light intensities of 250 ± 25 μmol photons m^-2^ s^-1^ at a 16:8 h light:dark cycle. In order to determine the carbonate chemistry, pH was measured every other day using a WTW 3110 pH meter equipped with a SenTix 41 Plus pH electrode (Wissenschaftlich-Technische Werkstätten GmbH, Weilheim, Germany), which was calibrated prior to each measurement to the National Bureau of Standards (NBS) scale. The precision of pH measurements during the experiments was ±0.02 units. Cells were acclimated to the *p*CO_2_ treatments for at least 7 generations (i.e. >21 days) prior to each experiment.

For the low-light treatments, the same conditions as the nutrient-replete dilute batch conditions were applied, except that incident light intensities were reduced to 55 ± 5 μmol photons m^-2^ s^-1^. In these incubations, CO_2_ concentrations ranged between ca. 16 and 50 μmol L^-1^, according to *p*CO_2_ values of 380, 800 and 1200 μatm. Nitrogen-limited conditions were achieved in gently mixed continuous cultures [35]. Cultures were grown as chemostats with fixed dilution rates representing ~33% of maximum growth for each species, with 0.15 ± 0.01 d^-1^ for *A*. *fundyense* and 0.2 ± 0.01 d^-1^ for *S*. *trochoidea*, yielding nitrate concentrations below 0.8 μmol L^-1^ for both species. In these incubations, CO_2_ concentrations ranged between ca. 8 and 40 μmol L^-1^, according to *p*CO_2_ values of 220, 800 and 1000 μatm (*A*. *fundyense*), and 280, 590 and 770 μatm (*S*. *trochoidea*). Steady state was reached after 22–43 days of acclimation, and samples were taken during this phase over 4 consecutive sampling points with time intervals of 2–3 days. For more details on the setup of the continuous culture experiment we refer to Eberlein et al. [19].

### Sampling and Analyses

For total alkalinity (TA) analysis, 50 mL culture suspension was filtered over cellulose acetate syringe filters (0.45 μm pore size, Thermo Scientific, Waltham, Massachusetts, USA) and stored in gas tight borosilicate bottles at 3°C. Samples were then analyzed in duplicates using an automated TitroLine burette system (SI Analytics, Mainz, Germany) with a precision of ±13 μmol L^-1^. Certified Reference Materials (CRMs) supplied by A. Dickson (Scripps Institution of Oceanography, USA) were used to correct for inaccuracies of TA measurements. TA was measured at the beginning and the end of each experiment, and during steady-state conditions in the continuous cultures. Minor changes in TA over the course of the experiments combined with the pH measurements every other day allowed for a complete resolution of the carbonate chemistry. The carbonate chemistry was assessed with the program CO2sys [36] using TA and pH (following recommendations of Hoppe et al. [37]) as well as temperature, salinity and phosphate concentration. We used the dissociation constants of carbonic acid and sulfuric acid of Mehrbach et al. [38], refitted by Dickson and Millero [39] and Dickson [40], respectively.

Duplicate samples of 20 mL culture suspension were fixed with neutral Lugol’s solution (2% final concentration) and counted every day or every other day with an inverted light microscope (Axiovert 40C, Zeiss, Germany). Growth rates during the exponential phase of growth were assessed separately for each biological treatment by fitting an exponential function through the cell numbers over time according to:

$$N=N_{0}e^{\mu t}$$ (1)

with *N* referring to cell number per mL at time *t* in days, *N*_*0*_ to the cell number per mL at the start of the experiment, and μ referring to the specific growth rate (d^-1^).

At the end of the experiment, when cells where still in exponential growth, we took samples to analyze Chl-a, POC and its isotopic composition (δ^13^C_POC_). For the analysis of Chl-a, duplicate samples of 200 mL of culture suspension were filtered over cellulose acetate filters (Whatman, Maidstone, UK). Filters were rapidly frozen in liquid nitrogen and stored at -80°C. Chl-a was extracted using 90% acetone with subsequent sonification for 0.5 min. Fluorescence was assessed using a TD-700 Fluorometer (Turner Designs, Sunnyvale, CA), and Chl-a concentrations were calculated according to Knap et al. [41]. To measure POC and PON quota and δ^13^C_POC_, 300–400 mL of culture suspension was filtered over pre-combusted GF/F filters (6 h, 500°C). Filters were stored in pre-combusted glass Petri dishes and 200 μL of HCl (0.2 mol L^-1^) was added to remove any inorganic carbon before they were dried overnight and stored at -25°C. POC quota and δ^13^C_POC_ of dilute batch experiments were then measured in duplicate with an Automated Nitrogen Carbon Analyser mass spectrometer (ANCA- SL 20–20, SerCon Ltd., Crewe, UK), with a precision of ± 0.5 μg C and 0.3‰, respectively. POC and PON quota and δ^13^C_POC_ of the continuous cultures were measured with a Delta S (Thermo) isotopic ratio mass spectrometer connected to an elemental analyzer CE1108 via an open split interface (Finnigan Conflow II). δ^13^C_POC_ is reported relative to the Vienna PeeDee Belemnite standard (VPDB). μ_c_ was calculated by multiplying μ with POC quota.

For isotopic measurements of the dissolved inorganic carbon (δ^13^C_DIC_), 4 mL of culture suspension was sterile filtered over 0.2 μm cellulose acetate filters (Thermo Scientific, Waltham, Massachusetts, USA) and stored at 3°C. 0.7 mL of the filtrate was then transferred to 8 mL vials, which contained three drops of 102% H_3_PO_4_ solution, and headspaces filled with helium. After equilibration, the isotopic composition in the headspace was measured using a GasBench-II coupled to a Thermo Delta-V advantage isotope ratio mass spectrometer, with a precision of ±0.1‰. ε_p_ was calculated relative to the isotopic composition of dissolved CO_2_ in the water (δ^13^C_CO2_) with an equation modified after Freeman and Hayes [42]:

$$\varepsilon_{p}=\;\frac{\delta^{13}{\text{C}}_{\text{CO}2}-\delta^{13}{\text{C}}_{\text{POC}}}{1+\frac{\delta^{13}{\text{C}}_{\text{POC}}}{1000}}$$ (2)

In order to calculate the isotopic composition of CO_2_ (δ^13^C_CO2_) from δ^13^C_DIC_, we calculated the isotopic composition of HCO_3_^-^ (δ^13^C_HCO3-_) based on δ^13^C_DIC_ according to a mass balance relation following Zeebe and Wolf-Gladrow [43] and the temperature-dependent fractionation factors between CO_2_ and HCO_3_^-^ and CO_3_^2-^ and HCO_3_^-^, as determined by Mook et al. [44] and Zhang et al. [45], respectively. For further details on the determination of carbon isotope fractionation we refer to Van de Waal et al. [25].

### Statistical analysis

Shapiro-Wilk tests confirmed normality of the data. Linear regressions were used to determine the relations between the tested variables and CO_2_. Significant differences between CO_2_ treatments were confirmed by one-way ANOVA followed by post hoc comparison of the means using the Tukey HSD (α = 0.05). A covariance analysis (ANCOVA) was used to determine homogeneity of slopes. When slopes were significantly different, i.e. when there were interactive effects of CO_2_ with light or nitrogen, the Johnson-Neyman technique (J-N; Johnson and Neyman [46]) was applied to identify the range of CO_2_ over which the investigated parameter was different. To improve the homogeneity of variances, as tested by Levene’s test, we used log_10_ transformed data for analysis of POC quota, μ_c_ and Chl-a:POC ratios of *G*. *spinifera*, and for analysis of Chl-a:POC and ε_p_ of *S*. *trochoidea*.

## Results

### Elevated *p*CO_2_ and light availability

**Fig 1:**
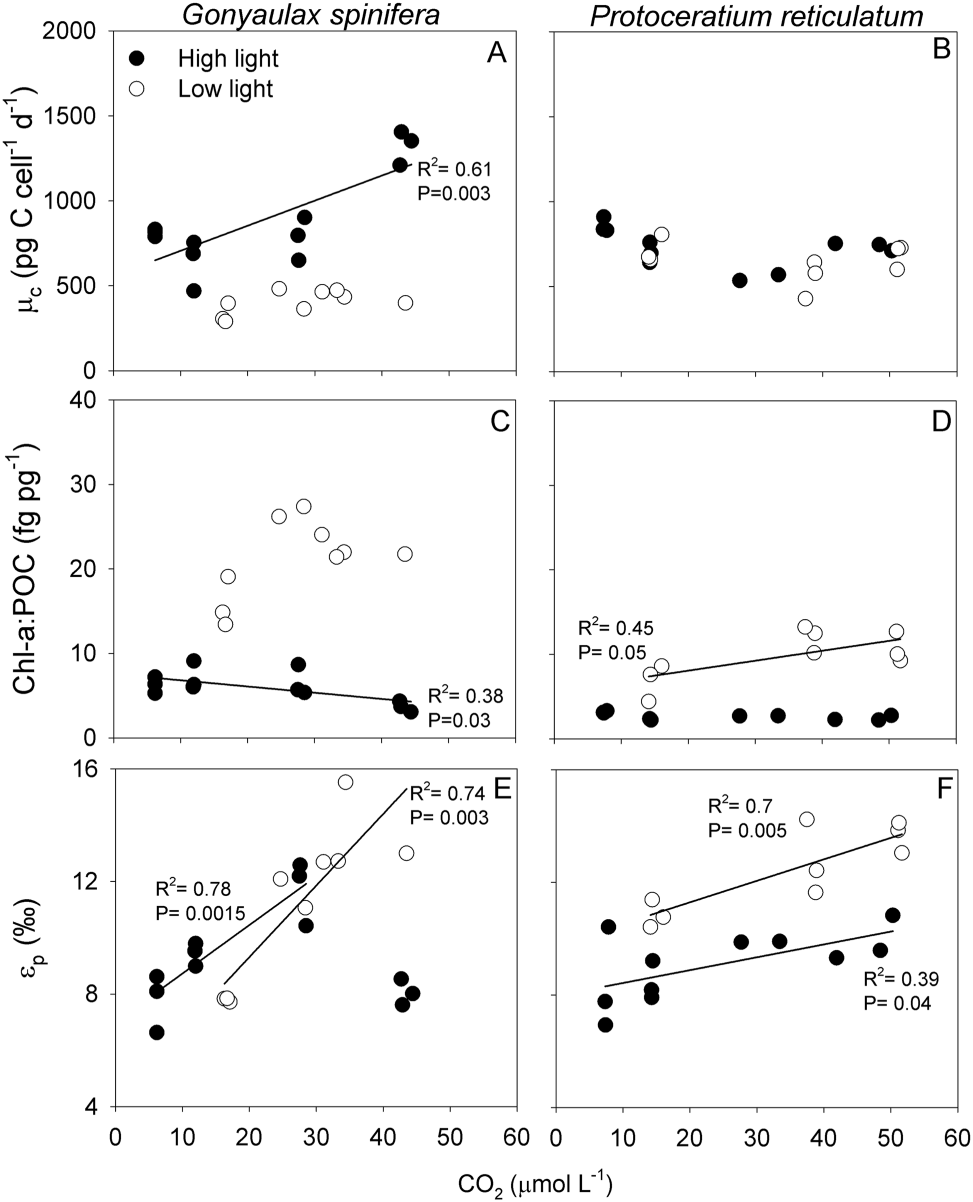
Combined effect of elevated pCO_2_ and light-limitation. (A, B) POC production, (C, D) Chl-a:POC ratios and (E, F) ε_p_ versus CO_2_ of *G*. *spinifera* (left) and *P*. *reticulatum* (right). Linear trend lines, R^2^ and P-values represent statistically significant relationships. Symbols indicate means of technical replicates. Means ± SD for all treatments are provided in Table 1. Note that the trend line for *G*. *spinifera* under HL excludes the highest *p*CO_2_ treatment (see also [16]). ε_p_ in the HL treatments have previously been published in Hoins et al. 2015.

In *G*. *spinifera*, μ_c_ did not change with CO_2_ availability under LL, but increased under HL, which was mainly driven by increased POC quota in the highest CO_2_ treatment (Fig 1A; linear regression; R^2^ = 0.60; P = 0.003) (see also [17]). Moreover, μ_c_ was lower under LL (ANCOVA; P<0.001; 95% CI [-0.635; -1.026]), which was due to decreased POC quota in all CO_2_ treatments, and due to lowered μ in all but the highest CO_2_ treatment (Table 1). In *P*. *reticulatum*, μ_c_ was not affected by CO_2_ under either LL or HL. Additionally, there was no interactive effect of CO_2_ and light availability on μ_c_. POC quota was unaffected by light in *P*. *reticulatum*, and significantly lower under LL in *G*. *spinifera* (ANCOVA; P<0.001; 95% CI [-1.024; 0.491]).

**Table 1:** Overview of the growth parameters in the HL and LL treatments. Growth rate (μ, d^-1^), POC quota (pg C cell^-1^), Chl-a content (pg cell^-1^) and ^13^C fractionation ε_p_ (‰) of *G*. *spinifera* and *P*. *reticulatum* grown under high-light and low-light conditions. Values represent the mean of triplicate incubations (n = 3 ±SD). Superscript letters indicate significant differences between *p*CO_2_ treatments (P<0.05). Superscript symbols refer to earlier published data in Hoins et al. 2015 (*).

*p*CO_2_ μatm	μ d^-1^	POC quota pg C cell^-1^	Chl a pg cell^-1^	ε_p_ ‰
*G*. *spinifera <LL>*				
380	0.19±0.03^a^	1743±271^a^	27.8±8.7^a^	7.8±0.1^a^
800	0.20±0.01^a^	2572±227^b^	66.2±3.3^b^	11.9±0.8^b^
1200	0.19±0.02^a^	2224±221^ab^	48.2±4.3^c^	13.7±1.5^b^
*G*. *spinifera <HL>*				
180	0.22±0.02^a,^*	3708±366^a,^*	23.1±2.4^a^	7.8±1.0^a,^*
380	0.23±0.01^a,^*	2758±583^a,^*	19.1±1.7^a^	9.4±0.4^a,^*
800	0.23±0.04^a,^*	3521±263^a,^*	22.1±2.3^a^	11.7±0.7^b,^*
1200	0.15±0.01^b,^*	8842±1044^b,^*	32.6±6.0^b^	8.0±0.5^a,^*
*P*. *reticulatum <LL>*				
380	0.25±0.01^a^	2843±233^a^	19.8±7.6^a^	10.9±0.5^a^
800	0.24±0.01^a^	2256±436^a^	26.6±3.9^b^	12.8±1.3^ab^
1200	0.27±0.01^a^	2552±204^a^	26.9±2.8^b^	13.7±0.6^b^
*P*. *reticulatum <HL>*				
180	0.28±0.00^a,^*	3099±119^a,^*	9.7±0.3^a^	8.4±1.8^a,^*
380	0.28±0.01^a,^*	2494±356^ab,^*	5.7±0.9^a^	8.4±0.7^a,^*
800	0.29±0.02^a,^*	2351±694^b,^*	5.5±0.4^a^	8.6±2.3^a,^*
1200	0.29±0.03^a,^*	2600±316^ab,^*	6.2±0.7^a^	9.9±0.8^a,^*

Ratios of Chl-a:POC increased with CO_2_ in *P*. *reticulatum* under LL (Fig 1D; linear regression; R^2^ = 0.45; P = 0.05), and were higher under LL for both *G*. *spinifera* and *P*. *reticulatum* (Fig 1C and 1D; ANCOVA; P<0.001; 95% CI [1.5; 1.1] and P<0.001; 95% CI [1.4; 1], respectively). Moreover, CO_2_ and light availability showed interactive effects on the Chl-a:POC ratios (ANCOVA; F_1,20_ = 9.453; P = 0.007 and F_1,19_ = 9.149; P = 0.008, respectively). In other words, the effect of CO_2_ depended on the light availability, with *P*. *reticulatum* showing a significant increase in Chl-a:POC ratios with CO_2_ availability under LL only (linear regression; R^2^ = 0.45; P = 0.05). Similarly, under LL Chl-a:POC ratios were significantly higher in the higher *p*CO_2_ treatments of *G*. *spinifera* (ANOVA; P<0.05).

Under LL, ε_p_ increased with CO_2_ in both *G*. *spinifera* and *P*. *reticulatum* (Fig 1E and 1F; linear regression; R^2^ = 0.74; P = 0.003 and R^2^ = 0.70; P = 0.005). Similar trends were observed under HL in *P*. *reticulatum* (linear regression; R^2^ = 0.39; P = 0.04) and, for CO_2_ levels between 180 and 800 μatm, also for *G*. *spinifera* (linear regression; R^2^ = 0.79; P = 0.001; see also [16]). CO_2_ and light showed interactive effects on ε_p_ in *G*. *spinifera* (ANCOVA; F_1,20_ = 10.968; P = 0.004), and ε_p_ of cells grown under LL versus HL were only significantly different in the highest CO_2_ treatment (>26; J-N; R^2^ = 0.56; P = 0.02; Fig 1E). In *P*. *reticulatum*, low-light resulted in higher ε_p_ across the tested CO_2_ concentrations (ANCOVA; P<0.001; 95% CI [1.7; 3.6]), with an average offset of 2.7‰.

### Elevated *p*CO_2_ and nitrogen-limitation

**Fig 2:**
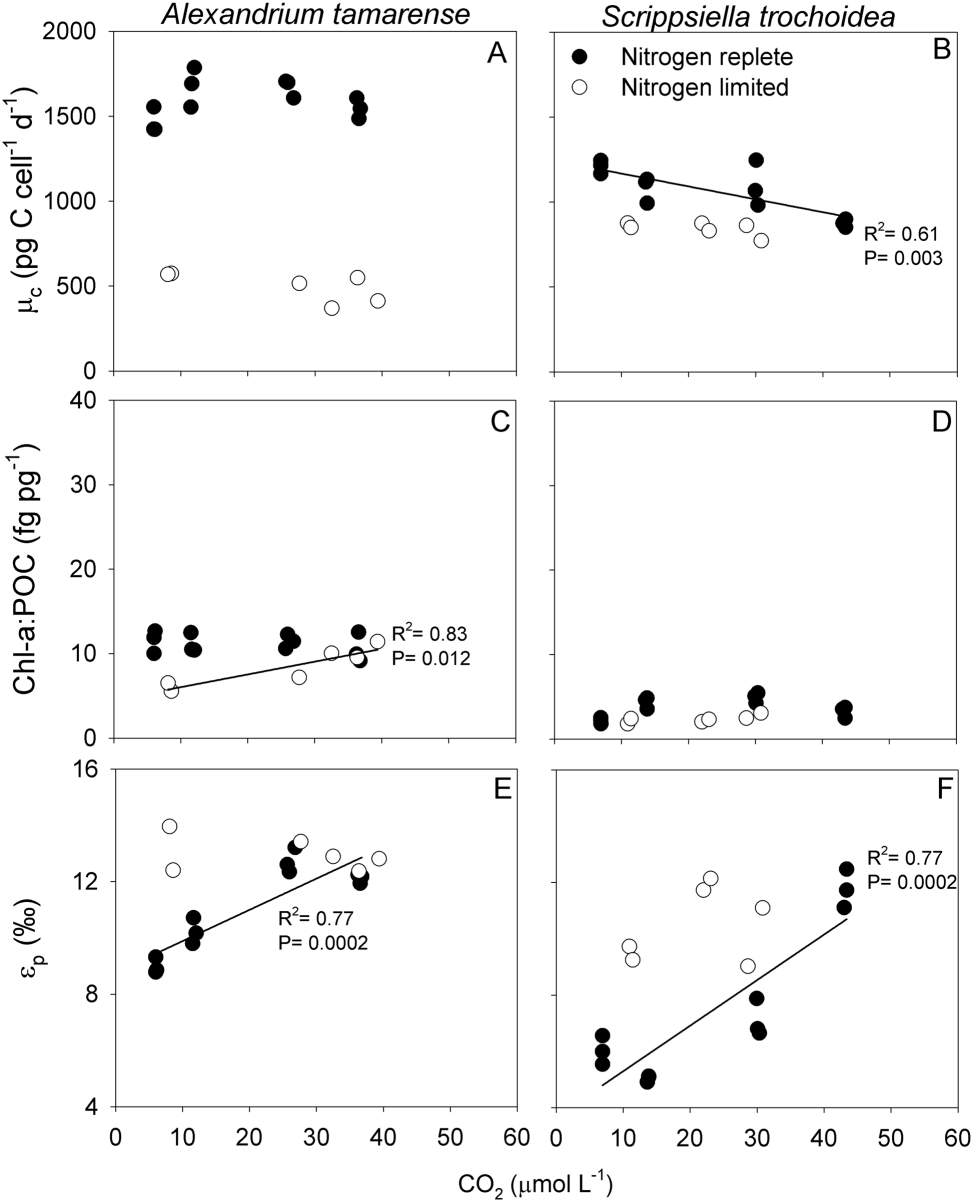
Combined effect of elevated pCO_2_ and nitrogen-limitation. (A, B) POC production, (C, D) Chl-a:POC ratios and (E, F) ε_p_ versus CO_2_ of *A*. *fundyense* (left) and *S*. *trochoidea* (right) cultured under nitrogen-replete conditions (HN; filled symbols) and nitrogen-limited conditions (LN; open symbols). Linear trend lines, R^2^ and P-values represent statistically significant relationships. Symbols indicate means of technical replicates. Means ± SD for all treatments are provided in Table 2. POC production and Chl-a:POC ratios have previously been published in [15] and [18], and ε_p_ in the HN treatments in Hoins et al. 2015.

In *A*. *fundyense*, μ_c_ did not change with CO_2_ when grown under either LN or HN. In *S*. *trochoidea*, μ_c_ was also independent of CO_2_ under LN, while it decreased with CO_2_ under HN (Fig 2B; linear regression; R^2^ = 0.61; P = 0.003). In both *A*. *fundyense* and *S*. *trochoidea*, μ_c_ was lowered under LN, independent of the CO_2_ concentration (Fig 2A and 2B; Table 2; ANCOVA; P<0.001; 95% CI [960; 1198] and P<0.001; 95% CI [-143; -321], respectively). LN did not affect POC quota in *A*. *fundyense*, but resulted in higher POC quota in *S*. *trochoidea* (ANCOVA; P<0.001; 95% CI [2591; 2261]).

**Table 2:** Overview of the growth parameters in the HN and LN treatments. Growth rate (μ, d^-1^), POC quota (pg C cell^-1^), Chl-a (pg cell^-1^), POC:PON ratios (molar) and ε_p_ (‰) of *A*. *fundyense* and *S*. *trochoidea* grown under nitrogen-replete conditions and nitrogen-limitation. Values represent the mean of duplicate incubations (n = 2 ±SD). Superscript letters indicate significant differences between *p*CO_2_ treatments (ANOVA; P<0.05; only applied when n>2). Superscript symbols refer to earlier published data in Hoins et al. 2015 (*), and Eberlein et al. 2014 (†) and 2016 (‡).

*p*CO_2_ μatm	μ d^-1^	POC quota pg C cell^-1^	Chl-a pg cell^-1^	POC:PON molar	ε_p_ ‰
*A*. *fundyense <LN>*					
220	0.15±0.01^a‡^	3930±212^a,‡^	22.9±2.0^a,‡^	9.52±0.46^a,‡^	13.18±1.1^a^
800	0.15±0.01^a‡^	2709±253^b,‡^	24.7±0.6^a,‡^	6.75±0.16^b,‡^	13.15±0.4^a^
1000	0.15±0.01^a‡^	3544±187^c,‡^	33.0±2.4^b,‡^	5.77±0.33^b,‡^	12.59±0.3^a^
*A*. *fundyense <HN>*					
180	0.46±0.02^a,b,†^	3169±254^a,†^	36.3±1.5^a,†^	5.76±0.1^a,†^	9.0±0.3^a,^*
380	0.46±0.02^ab,†^	3620±308^a,†^	40.1±2.8^a,†^	5.77±0.3^a,†^	10.2±0.5^b,^*
800	0.48±0.01^a,†^	3455±153^a,†^	39.5±3.3^a,†^	5.73±0.1^a,†^	12.7±0.4^c,^*
1200	0.45±0.01^b,†^	3461±165^a,†^	36.4±5.8^a,†^	5.6±0.1^a,†^	12.1±0.2^c,^*
*S*. *trochoidea <LN>*					
280	0.2±0.01^a‡^	4292±243^a,‡^	9.0±1.3^a,‡^	21.3±1.3^a,b,‡^	9.5±0.3^a^
590	0.2±0.01^a‡^	4239±220^a,‡^	9.2±0.6^a,‡^	24.7±1.6^b,‡^	11.9±0.3^a^
770	0.2±0.01^a‡^	4065±254^a,‡^	11.2±0.9^b,‡^	18.0±0.9^a,‡^	10.1±1.5^a^
*S*. *trochoidea <HN>*					
180	0.61±0.03^a,†^	1990±36^a,†^	4.3±0.7^a,†^	7.6±0.2^ac,†^	6.0±0.5^ab,^*
380	0.61±0.05^a,†^	1762±15^ab,†^	7.6±1.2^ab,†^	8.1±0.3^ab,†^	5.0±0.1^a,^*
800	0.61±0.04^a,†^	1787±223^ab,†^	8.7±0.5^b,†^	8.4±0.3^b,†^	7.1±0.7^b,^*
1200	0.58±0.02^a,†^	1500±85^b,†^	4.9±1.3^a,†^	7.4±0.1^c,†^	11.8±0.7^c,^*

Chl-a:POC ratios were interactively affected by CO_2_ and nitrogen availability in *A*. *fundyense* (ANCOVA; F_1,17_ = 13.393; P = 0.003), and cells grown under LN showed lower ratios at low CO_2_ concentrations (i.e. <30 μmol L^-1^; J-N; R^2^ = 0.73; P<0.001). In *S*. *trochoidea*, Chl-a:POC ratios were slightly lower under LN at all tested CO_2_ concentrations (ANCOVA; P = 0.041; 95% CI [0.02; 0.6]). Under LN, POC:PON ratios were significantly higher in *S*. *trochoidea* in all tested *p*CO_2_ treatments and in the lowest *p*CO_2_ treatment of *A*. *fundyense* (ANOVA: P<0.05). POC:PON ratios were significantly lowered in the higher *p*CO_2_ treatments of both species (ANOVA; P<0.05; Table 2) [19].

Under LN, ε_p_ was independent of CO_2_ in both *A*. *fundyense* and *S*. *trochoidea* (Fig 2E and 2F), while there were positive correlations under HN (Fig 2E and 2F; linear regression; R^2^ = 0.76; P<0.001 and R^2^ = 0.77; P<0.001, respectively; see also [17]). In *A*. *fundyense*, CO_2_ and nitrogen availability showed interactive effects on ε_p_ (ANCOVA; F_1,17_ = 17.359; P = 0.001), with significantly higher ε_p_ values at lower CO_2_ concentrations (i.e. <29 μmol L^-1^; J-N; R^2^ = 0.82, P<0.001). When grown under LN, both species show a relatively constant ε_p_ of around 13.0±0.6‰ in *A*. *fundyense* and 10.5±1.3‰ in *S*. *trochoidea*. These values are similarly high as the highest ε_p_ values obtained in the dilute batch cultures under HN (12.4±0.4 and 11.8±0.7‰, respectively).

## Discussion

### Production rates, quotas and stoichiometry

Our results show differential effects of elevated *p*CO_2_ in combination with light availability on growth, POC quota, μ_c_ and Chl-a:POC ratios in *G*. *spinifera* and *P*. *reticulatum* (Fig 1; Table 1). In *G*. *spinifera*, μ_c_ increased with CO_2_ under HL, but there was no sensitivity towards elevated *p*CO_2_ under LL (Fig 1A). Low-light furthermore caused lowered POC quota and μ_c_, while μ remained unaffected (Fig 1A and 1B; Table 1). At the same time, Chl-a contents and Chl-a:POC ratios increased under LL (Fig 1C and 1D; Table 1). Such higher ratios are needed to capture more light, which is a general response of phytoplankton to light-limitation. For *P*. *reticulatum*, the low light conditions did not yield changes in POC quota, μ and μ_c_ (Fig 1A and 1B; Table 1). This suggests a high flexibility of *P*. *reticulatum* to deal with low-light conditions. Cells did synthesize more Chl-a, thereby showing elevated Chl-a:POC ratios, which were apparently sufficient to compensate for the low-light conditions. Both species showed increasing Chl-a:POC ratios with increasing CO_2_ availability when grown under low-light. This suggests that CO_2_ influences the ability of cells to synthesize Chl-a, and therefore their ability to cope with low-light conditions.

We observed generally minor effects of elevated *p*CO_2_ under LN, while nitrogen- limitation alone exerted a much stronger control (Fig 2, Table 2). Specifically, μ_c_ was lower in both *A*. *fundyense* and *S*. *trochoidea*, although POC quota in *S*. *trochoidea* grown under LN was significantly higher. Decreased μ_c_ was mainly a result of low μ, i.e. the imposed dilution rate which was set at about 33% of the μ of the respective species obtained from experiments under replete conditions. Nitrogen-limitation was confirmed by the higher POC:PON ratios in *S*. *trochoidea* in all tested *p*CO_2_ treatments, while POC:PON ratios of *A*. *fundyense* grown under LN were only higher in the lowest *p*CO_2_ treatment (Table 2) [19]. The Chl-a:POC ratios in LN were comparable to HN in *S*. *trochoidea*, while in *A*. *fundyense* these Chl-a:POC ratios showed a CO_2_-dependent increase under LN, and only differed between HN and LN under low CO_2_ concentrations. Thus, although nitrogen was limiting μ_c_ and caused an increase in POC:PON ratios, this did not strongly affect the Chl-a:POC ratios.

Irrespective of the light intensity or nitrogen concentration, CO_2_ effects on growth rates, POC quotas and POC production in our study were either absent or relatively minor, suggesting the presence of effective carbon concentrating mechanisms (CCMs). Dinoflagellates possess RubisCO type II with lowest CO_2_ affinities compared to all other eukaryotic algae [47; 48], which make effective CCMs a prerequisite to maintain growth under low CO_2_ concentrations. Indeed, earlier work has shown that *A*. *fundyense* and *S*. *trochoidea* are able to actively take up HCO_3_^-^, thus increasing their intracellular C_i_ pool [16]. Additionally, high extracellular activities of carbonic anhydrase, the enzyme accelerating the otherwise slow interconversion between CO_2_ and HCO_3_^-^, have been found in *S*. *trochoidea* [16]. Consequently, at least the investigated dinoflagellate species do not seem to be CO_2_-limited in any of tested CO_2_ concentrations, irrespective of the light or nutrient supply, explaining why μ, POC quotas and μ_c_ did not respond to elevated CO_2_ concentrations.

In the cyanobacterium *Trichodesmium* and the coccolithophore *Emiliania huxleyi*, limitation by light has been shown to cause enhanced sensitivity towards elevated *p*CO_2_ [49; 50]. The CO_2_-dependent stimulation of μ_c_ was most pronounced under light-limitation, which was explained by larger CO_2_-dependent benefits due to the CCM down-regulation and thus energy reallocation under light-limitation. In the tested dinoflagellate species, however, μ_c_ remained largely unaltered over the applied CO_2_ range (Figs 1A, 1B, 2A and 2B). Yet, we observed a CO_2_-dependent increase in Chl-a:POC quota in *G*. *spinifera* and *P*. *reticulatum* grown under low-light. Thus, with elevated *p*CO_2_ more energy is acquired via photosynthesis, while the same level of μ_c_ is maintained. It is further conceivable that their CCMs are down-regulated with elevated *p*CO_2_, lowering the energetic costs for carbon acquisition. The likely higher availability of energy with elevated *p*CO_2_ under low-light conditions, however, seems not to be allocated to μ_c_ (Figs 1C, 1D and 2C). This suggests either a lower overall efficiency to convert energy to biomass under these conditions, or a shunting of energy to alternative processes not accounted for in our study. Similarly to the Chl-a:POC ratios in *G*. *spinifera* and *P*. *reticulatum* under low-light conditions, Chl-a:POC ratios in *A*. *fundyense* grown under nitrogen-limitation also increased at elevated CO_2_ concentrations. When grown under nitrogen-limitation, excess energy from a down-regulation of CCMs may be shunted to nitrogen acquisition. Indeed, POC:PON ratios decreased under elevated *p*CO_2_ for both *A*. *fundyense* and *S*. *trochoidea* (Table 2) (see also [19]). Such lower POC:PON ratios (i.e. relatively more nitrogen) may favor synthesis of nitrogen-rich biomolecules such as Chl-a. Overall, elevated *p*CO_2_ seems to have only minor effects on growth and μ_c_ in the tested dinoflagellates, and yet it apparently causes intracellular shifts in energy and resource allocation under light- or nitrogen-limited conditions.

### ^13^C fractionation

**Fig 3:**
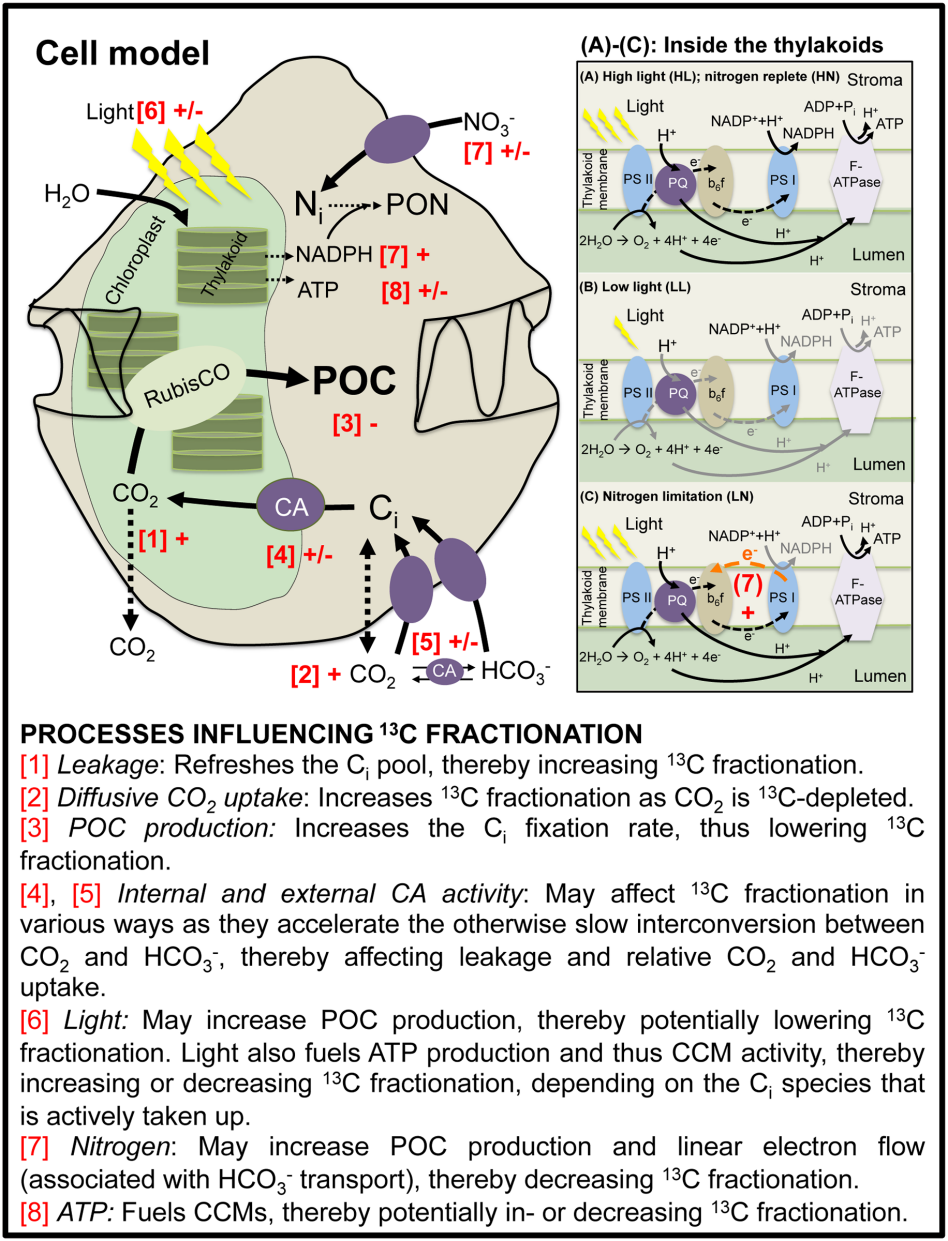
Conceptual model of a dinoflagellate cell and processes at the thylakoid membrane of the chloroplasts. (A) high-light (HL) and nitrogen-replete (HN) conditions, (B) low-light conditions (LL) and (C) nitrogen-limitation (LN). Processes potentially influencing ^13^C fractionation ([1]–[8]) are highlighted in red, while + and − refer to an increase or decrease in ^13^C fractionation, respectively. (A) Saturating light and nutrient-replete conditions: Light provides the energy (= photons) needed for Photosystem II (PSII; in thylakoid membrane) to oxidize water to O_2_, thereby producing electrons (e^-^) and protons (H^+^). Electrons are transported by plastohydroquinone (PQ), thereby pumping more protons into the lumen. The cytochrome b_6_f complex oxidizes PQ molecules, thereby producing electrons, which are then transported to Photosystem I (PSI) where they reduce NADP^+^ to NADPH. Protons are transported to F-ATPase to synthesize ATP. (B) Under light-limitation, the overall decreased amount of energy arriving at PSII causes a decrease in water oxidation, thereby producing less electrons and protons, and thus also less ATP and NADPH. (C) Under nitrogen-limitation, less NADPH is needed for NO_3_^-^ reduction, thus the excess electrons are transported back to PSII by cyclic energy flow. Protons are still pumped by F-ATPase, thereby increasing the amount of ATP synthesized.

The ^13^C fractionation of phytoplankton is influenced by the interplay between 1) CO_2_ supply, 2) inorganic carbon demand (i.e. μ_c_), and 3) active uptake of CO_2_ and HCO_3_^-^ (i.e. CCMs). If CO_2_ supply in the growth medium increases, ε_p_ increases because more of the ^13^C-depleted CO_2_ may be taken up in comparison to the ^13^C-enriched HCO_3_^-^. In contrast, ε_p_ may decrease with increasing μ_c_ as CO_2_ is fixed at a higher rate than total carbon is taken up, and the ability of RubisCO to express its full preference for ^12^CO_2_ is reduced. CCMs can influence ε_p_ in various ways, e.g. as they determine the relative uptake of CO_2_ and HCO_3_^-^ as well as leakage (Fig 3). Under HL and HN conditions, ε_p_ shows a clear increase with increasing CO_2_ concentrations in all four tested dinoflagellate species ([17]; Figs 1 and 2). Under LL, similar CO_2_ dependencies were observed, although ε_p_ shifted to higher values in *P*. *reticulatum*. Under LN, ε_p_ was not CO_2_ sensitive, and remained relatively high also at lower CO_2_ concentrations for both *A*. *fundyense* and *S*. *trochoidea*.

Light- or nutrient-limitation cause changes in the availability of energy (ATP) and reductants (NADPH) that in turn may affect μ_c_ and CCM activity, eventually influencing ε_p_ (Fig 3). Under low-light conditions, for instance, less photons arrive at the photosystems, thereby lowering the H_2_O splitting and thus the production of electrons and protons (Fig 3B). The lowered electron and proton fluxes then result in lower amounts of ATP and NADPH. ATP is required to operate the energetically costly CCMs, while both ATP and NADPH are required for CO_2_ reduction in the Calvin Cycle to produce biomass, and for reducing nitrate (NO_3_^-^) to ammonium (NH_4_^+^) to eventually produce particulate organic nitrogen (PON). Thus, differences in the availability of light but also nitrogen alter the availability of ATP and NADPH, which may be one reason for the differences in ε_p_ responses between types of incubations (e.g. [27; 51; 52; 30]).

High-light intensities may provide the cells with more energy than required for CO_2_ fixation, which will enhance the active uptake of C_i_ that in turn serves as an energy sink for excess light [53]. Depending on how much C_i_ is taken up in relation to the amount of CO_2_ that is fixed, a high C_i_ uptake may be accompanied by a high leakage [54]. A high C_i_ uptake by *G*. *spinifera* at both LL and HL, in concert with high leakage, would explain its relatively high ε_p_. In contrast to our expectations, however, ε_p_ in *P*. *reticulatum* was substantially lower under HL conditions. In this species, an increasing contribution of energetically costly HCO_3_^-^ uptake under HL may support the dissipation of excess energy, avoiding damage to photosystem II. If this active HCO_3_^-^ uptake does not lead to higher leakage, it could in fact explain the lower ε_p_ under HL.

Comparable to light, also nitrogen availability may alter ε_p_ as it indirectly changes cellular energy budgets (Fig 3). As mentioned, NADPH is used to reduce CO_2_ to organic carbon, and NO_3_^-^ to NH_4_^+^. As a consequence, less NADPH is needed when μ_c_ is low and/or when NO_3_^-^ is limiting. Under these conditions, cyclic electron flow “around” photosystem I may be up-regulated, thereby circumventing NADPH production while maintaining ATP generation (Fig 3C). Such a putative excess of ATP over NADPH, in turn, may be used for active inorganic carbon uptake. As ε_p_ in both *A*. *fundyense* and *S*. *trochoidea* was higher under nitrogen- limitation, CO_2_ and not HCO_3_^-^ may have been taken up actively. Alternatively, increasing overall C_i_ uptake despite low μ_c_ may have increased leakage and thus ε_p_. Nonetheless, even the highest ε_p_ of ~14‰ in our study was low compared to earlier studies investigating the effect of nitrogen-limitation on ε_p_ in other algal species [27; 51; 28; 55]. This is in line with the generally high uptake of HCO_3_^-^ observed in earlier studies on CCMs in dinoflagellates [14; 16]. Moreover, maximum ^13^C fractionation of RubisCO in the tested dinoflagellate species may be lower than the typical 24–30‰, as was also found for a RubisCO isolated from *E*. *huxleyi* (i.e. 11‰; [56]).

### Proxy development

The CO_2_-dependency of ε_p_ in dinoflagellates can potentially serve in the development of a proxy for past *p*CO_2_ in the atmosphere [17]. As indicated before, however, additional experiments focusing on environmental variables other than *p*CO_2_, physiological underpinning of the recorded response, quantification of fractionation between dinoflagellate cells and cysts, as well as field calibration studies are required to establish a reliable proxy [17]. Here, we investigated the possible role of environmental variables other than *p*CO_2_, including light and nitrogen availability.

The results show that under low-light conditions, the general response of ε_p_ towards elevated *p*CO_2_ remains largely unaltered in *G*. *spinifera* and *P*. *reticulatum*, i.e. slopes remained largely similar. In contrast, ε_p_ becomes insensitive to changes in CO_2_ under nitrogen-limitation in *A*. *fundyense and S*. *trochoidea*. Elevated *p*CO_2_ in the past was presumable accompanied by water column stratification, thereby not only affecting the water depth at which dinoflagellates fixed carbon, but also the potential upwelling of nutrient-rich deeper water masses. Consequently, it is crucial to take into account the light conditions and nutrient concentrations during the dinoflagellate lifetime.

Application of an eventual proxy based on dinoflagellate ε_p_ would likely be most valuable at study sites where nitrate concentrations are non-limiting and stable through time. For such settings, the equilibrium between dissolved (recorded in dinoflagellates) and atmospheric (the proxy target) *p*CO_2_ is typically sub-optimal. This results in an interesting paradox since study sites are required for which CO_2_ is equilibrated between the ocean and atmosphere, and also bear sufficient nutrients to force a CO_2_ response in ε_p_. Moreover, intense blooms of dinoflagellates may deplete seawater not only in CO_2_ [57; 58], but also in nutrients, leading to a potential bias in ε_p_.

Thus, although ε_p_ shows largely consistent CO_2_ dependencies across four tested dinoflagellate species under optimal growth conditions [16], other environmental factors, notably nitrogen limitation, complicate and possibly negate the suitability of dinoflagellate ε_p_ as a proxy for past pCO_2_.

## Supporting Information

S1 Appendix Overview of the carbonate chemistry in all treatments. Average dissolved CO_2_ concentrations (μmol L^-1^), total alkalinity (TA: μmol L^-1^), dissolved inorganic carbon (DIC; μmol L^-1^) and pH (NBS scale). Values represent the mean (±SD) of triplicate incubations (n = 3), except for LN experiments which represent the mean of duplicate incubations (n = 2 ±SD). Superscript letters indicate significant differences between *p*CO_2_ treatments (ANOVA; P<0.05, only applied when n>2).
(DOCX)
