# Supplementary material for: Combined Effects of Ocean Acidification and Light or Nitrogen Availabilities on 13C Fractionation in Marine Dinoflagellates
Source: PLoS One. 2016 May 6;11(5):e0154370. doi: 10.1371/journal.pone.0154370 (PMC4859495; doi:10.1371/journal.pone.0154370)
Supplement: S1 Appendix — Average dissolved CO2 concentrations (μmol L-1), total alkalinity (TA: μmol L-1), dissolved inorganic carbon (DIC; μmol L-1) and pH (NBS scale). Values represent the mean (±SD) of triplicate incubations (n = 3), except for LN experiments which represent the mean of duplicate incubations (n = 2 ±SD). Superscript letters indicate significant differences between pCO2 treatments (ANOVA; P<0.05, only applied when n>2). (DOCX) [file pone.0154370.s001.docx]

**Appendix 1.** Overview of the carbonate chemistry in all treatments. Average dissolved CO_2_ concentrations (μmol L^-1^), total alkalinity (TA: μmol L^-1^), dissolved inorganic carbon (DIC; μmol L^-1^) and pH (NBS scale). Values represent the mean (±SD) of triplicate incubations (n=3), except for LN experiments which represent the mean of duplicate incubations (n=2 ±SD). Superscript letters indicate significant differences between *p*CO_2_ treatments (ANOVA; P<0.05, only applied when n>2).

| *p*CO_2_  μatm | CO_2_  μmol L^-1^ | TA  μmol L^-1^ | DIC  μmol L^-1^ | pH  NBS |  | *p*CO_2_  μatm | CO_2_  μmol L^-1^ | TA  μmol L^-1^ | DIC  μmol L^-1^ | pH  NBS |
| --- | --- | --- | --- | --- | --- | --- | --- | --- | --- | --- |
| *G. spinifera <LL>* | | | | |  | *A. tamarense <LN>* | | | | |
| 380 | 16.7±0.9 | 2413±32 | 2427±5^a^ | 8.14±0.02^a^ |  | 220 | 8.4±0.4^[18]^ | 2372±11^[18]^ | 2099±8^a,[18]^ | 8.39±0.2^a,[18]^ |
| 800 | 28.1±3.0 | 2412±26 | 2399±17^b^ | 7.95±0.04^b^ |  | 800 | 30.1±3.4^[18]^ | 2398±21^[18]^ | 2253±13^b,[18]^ | 7.92±0.05^b,[18]^ |
| 1200 | 37.1±3.9 | 2407±1 | 2412±25^b^ | 7.84±0.04^c^ |  | 1000 | 37.9±2.1^[18]^ | 2397±7^[18]^ | 2274±13^b,[18]^ | 7.82±0.02^c,[18]^ |
| *G. spinifera <HL>* | | | | |  | *A. tamarense <HN>* | | | | |
| 180 | 6.4±1.0^[16]^ | 2447±5^[16]^ | 1962±15^a,[16]^ | 8.50±0.05^a,[16]^ |  | 180 | 6.1±0.9^[15;16]^ | 2434±3^[15;16]^ | 1992±33^a,[15;16]^ | 8.50±0.06^a,[15;16]^ |
| 380 | 12.4±2.2^[16]^ | 2461±12^[16]^ | 2083±1^b,[16]^ | 8.27±0.07^b,[16]^ |  | 380 | 11.8±2.1^[15;16]^ | 2439±1^[15;16]^ | 2117±41^b,[15;16]^ | 8.27±0.07^b,[15;16]^ |
| 800 | 29.3±6.7^[16]^ | 2475±13^[16]^ | 2224±9^c,[16]^ | 7.96±0.10^c,[16]^ |  | 800 | 26.5±5.8^[15;16]^ | 2434±2^[15;16]^ | 2245±37^c,[15;16]^ | 7.97±0.10^c,[15;16]^ |
| 1200 | 44.7±6.4^[16]^ | 2459±4^[16]^ | 2293±5^d,[16]^ | 7.78±0.06^d,[16]^ |  | 1200 | 37.3±9.3^[15;16]^ | 2418±1^[15;16]^ | 2283±34^d,[15;16]^ | 7.83±0.12^d,[15;16]^ |
| *P. reticulatum <LL>* | | | | |  | *S. trochoidea <LN>* | | | | |
| 380 | 14.8±1.9 | 2448±10 | 2160±4^a^ | 8.20±0.05^a^ |  | 280 | 11.2±0.4^[18]^ | 2349±6^[18]^ | 2148±0^a,[18]^ | 8.30±0.01^a,[18]^ |
| 800 | 38.4±5.7 | 2456±16 | 2307±6^b^ | 7.84±0.06^b^ |  | 590 | 22.6±0.7^[18]^ | 2389±5^[18]^ | 2229±2^b,[18]^ | 8.03±0.01^b,[18]^ |
| 1200 | 51.3±8.7 | 2471±20 | 2330±2^c^ | 7.73±0.07^b^ |  | 770 | 29.8±1.5^[18]^ | 2365±1^[18]^ | 2251±8^c,[18]^ | 7.92±0.02^c,[18]^ |
| *P. reticulatum <HL>* | | | | |  | *S. trochoidea <HN>* | | | | |
| 180 | 7.6±0.5^[16]^ | 2460±8^[16]^ | 2002±2^a,[16]^ | 8.43±0.04^a,[16]^ |  | 180 | 6.8±0.2^[15;16]^ | 2386±1^[15;16]^ | 1872±16^a,[15;16]^ | 8.45±0.01^a,[15;16]^ |
| 380 | 14.8±0.8^[16]^ | 2455±2^[16]^ | 2121±4^b,[16]^ | 8.21±0.02^b,[16]^ |  | 380 | 13.4±0.5^[15;16]^ | 2388±2^[15;16]^ | 2096±10^b,[15;16]^ | 8.21±0.02^b,[15;16]^ |
| 800 | 28.5±2.7^[16]^ | 2461±12^[16]^ | 2249±23^c,[16]^ | 7.88±0.08^c,[16]^ |  | 800 | 29.5±2.1^[15;16]^ | 2385±1^[15;16]^ | 2223±11^c,[15;16]^ | 7.91±0.03^c,[15;16]^ |
| 1200 | 43.0±3.4^[16]^ | 2473±19 | 2288±16^d,[16]^ | 7.75±0.05^d,[16]^ |  | 1200 | 42.5±3.6^[15;16]^ | 2386±4^[15;16]^ | 2268±18^d,[15;16]^ | 7.77±0.04^d,[15;16]^ |
